# Prevalence of orthostatic hypertension and its association with cerebrovascular diagnoses in patients with suspected TIA and minor stroke

**DOI:** 10.1186/s12872-022-02600-1

**Published:** 2022-04-09

**Authors:** Farzaneh Barzkar, Phyo K. Myint, Chun Shing Kwok, Anthony Kneale Metcalf, John F. Potter, Hamid Reza Baradaran

**Affiliations:** 1 Center for Educational Research in Medical Sciences, Faculty of Medicine, Iran University of Medical Sciences, IUMS, Tehran, Iran; 2 Norwich Medical School, University of East Anglia, Norwich, UK; 3 Stroke Research Group, Norwich Cardiovascular Research Group, Norwich Research Park, Norwich, UK; 4 School of Medicine, Keele University, Stoke-on-Trent, UK; 5 Stroke Services, Norfolk and Norwich University Hospitals, NHS Foundation Trust, Norwich, UK; 6 Endocrinology and Metabolism Research Center, Institute of Endocrinology and Metabolism, Iran University of Medical Sciences, Tehran, Iran; 7 Ageing Clinical and Experimental Research (ACER) Team, Institute of Applied Health Sciences, University of Aberdeen, Aberdeen, UK

**Keywords:** Stroke, Transient ischemic attack, Cerebrovascular disease, Orthostatic hypertension, Blood pressure

## Abstract

**Purpose:**

We aimed to compare the rate of stroke, transient ischemic attack, and cerebrovascular disease diagnoses across groups of patients based on their orthostatic blood pressure response in a transients ischemic attack clinic setting.

**Materials and Methods:**

We retrospectively analysed prospectively collected data from 3201 patients referred to a transient ischemic attack (TIA)/minor stroke outpatients clinic. Trained nurses measured supine and standing blood pressure using an automated blood pressure device and the patients were categorized based on their orthostatic blood pressure change into four groups: no orthostatic blood pressure rise, systolic orthostatic hypertension, diastolic orthostatic hypertension, and combined orthostatic hypertension. Then, four stroke physicians, who were unaware of patients' orthostatic BP response, assessed the patients and made diagnoses based on clinical and imaging data. We compared the rate of stroke, TIA, and cerebrovascular disease (either stroke or TIA) diagnoses across the study groups using Pearson's χ^2^ test. The effect of confounders was adjusted using a multivariate logistic regression analysis.

**Results:**

Cerebrovascular disease was significantly less common in patients with combined systolic and diastolic orthostatic hypertension compared to the "no rise" group [OR = 0.56 (95% CI 0.35–0.89]. The odds were even lower among the subgroups of patients with obesity [OR = 0.31 (0.12–0.80)], without history of smoking [OR 0.34 (0.15–0.80)], and without hypertension [OR = 0.42 (95% CI 0.19–0.92)]. We found no significant relationship between orthostatic blood pressure rise with the diagnosis of stroke. However, the odds of TIA were significantly lower in patients with diastolic [OR 0.82 (0.68–0.98)] and combined types of orthostatic hypertension [OR = 0.54 (0.32–0.93)]; especially in patients younger than 65 years [OR = 0.17 (0.04–0.73)] without a history of hypertension [OR = 0.34 (0.13–0.91)], and patients who did not take antihypertensive therapy [OR = 0.35 (0.14–0.86)].

**Conclusion:**

Our data suggest that orthostatic hypertension may be a protective factor for TIA among younger and normotensive patients.

**Supplementary Information:**

The online version contains supplementary material available at 10.1186/s12872-022-02600-1.

## Introduction

Globally, stroke is the second leading cause of adult mortality, morbidity and disability. The majority of these events occur in low to middle-income countries [1, 2]. Since treatment and rehabilitation after stroke are very costly, prevention strategies during the course of the disease are an important area of focus for stroke risk reduction. The most common modifiable risk factor of stroke is hypertension [3]. Indeed, disorders of orthostatic blood pressure response and increased diurnal BP variability have been shown to be associated with worse cardiovascular (CV) outcomes [4–7]. However, the direction and strength of this relationship is more clear for orthostatic hypotension and non-dipping or reverse- night-time-dipping pattern of diurnal BP variation compared to orthostatic hypertension (OHT) and an extreme-dipping pattern. A better understanding of blood pressure fluctuations and OHT may enable better patient care through a personalized approach.

OHT is defined as an increase in BP upon assuming an upright position [8]. It is clinically important as it has been shown to be a risk factor for lacunar stroke and a prognostic factor for mortality and frailty among the elderly [5, 7–9]. Whether this condition is a precursor to hypertension and whether postural rise in BP can affect the risk of stroke in patients without hypertension is unknown [5, 10]. On the other hand, some research has shown a protective effect of OHT and extreme-dipping on cerebrovascular disease [8, 11]. It is further unknown whether conditions associated with orthostatic blood pressure control are actually the cause or result of stroke or an association that happens in line with cerebrovascular ageing [7, 12–21]. Some studies have postulated an increased sympathetic response, baroreceptor sensitivity, baroreceptor aging, and increased humoral adrenergic concentrations as possible pathophysiologic mechanisms for the condition [9].

The cut-offs and definitions of OHT and whether systolic and diastolic OHT may be different pathophysiological entities are currently unknown. Most of existing literature have not divided different types and patterns of OHT in their assessments for cerebrovascular correlations. In this study, we aimed to investigate the association between different patterns of orthostatic hypertension and the clinical diagnosis of stroke, transient ischaemic attack (TIA), or no cerebrovascular diagnosis in a large cohort of patients assessed in a TIA clinic in the East of England.

## Methods

### Study design, setting, participants, study size

**Fig. 1:**
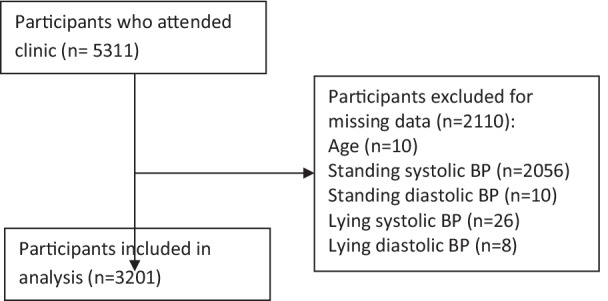
Flow diagram of participant inclusion

We retrospectively assessed prospectively-collected data from 3201 patients who presented to a TIA clinic at a University Hospital in the East of England (catchment population of ~ 750,000) between July 2002 and September 2009. The data were retrieved from a TIA database which is part of the Stroke & TIA Register that received Institutional approval from the local NHS Trust and ethical approval from Newcastle and North Tyneside Research Ethics Committee (ref: 17/NE/0277). Further information on the inclusion of patients is presented in Fig. 1.

### Classification of patients

The patients were classified into four categories based on their BP dynamics according to the most commonly used definition of Orthostatic Hypertension as an increase in BP of either/both**:** ≥ 20 mmHg systolic or ≥ 10 mmHg diastolic from lying to standing/sitting [10].

The main study groups were:

1. Systolic orthostatic hypertension (a rise in BP of ≥ 20 mmHg systolic from lying to standing/sitting regardless of DBP level).
2. Diastolic orthostatic hypertension (a rise in BP of ≥ 10 mmHg diastolic from lying to standing/sitting regardless of SBP level).
3. Combined orthostatic hypertension (a rise in BOTH ≥ 20 mmHg systolic and ≥ 10 mmHg diastolic from lying to standing/sitting).
4. Patients without significant orthostatic rise in BP referred to as the "No orthostatic hypertension" group who consisted of patients without orthostatic blood pressure change and patients with orthostatic hypotension.

### Variables and data sources/ measurement

At each clinic appointment, trained nurses measured supine and standing BP using a DINAMAP ® (GE Health Care) automated BP device, with a cuff of the appropriate size, (Easton JD, Saver JL, Albers GW et al.) prior to specialist stroke physician assessment (without the knowledge of final diagnosis). BP was recorded after 5 min of supine rest, and after standing for 3 min with feet on the floor. The sitting blood pressure reading was used for patients who were unable to stand. Age, gender, past medical history (prior stroke or TIA, co-morbidities, which included AF, hypertension, ischaemic heart disease, peripheral vascular disease (PVD), diabetes, hyperlipidaemia and obesity defined as calculated BMI of ≥ 30 kg/m^2^), smoking status (yes/no), alcohol consumption (yes/no) and type of antihypertensive medications (thiazide diuretics, ACEI/ARBs, b-blockers, spironolactone, other diuretics, calcium-channel blockers and alpha-blocker) were also collected. Stroke physicians would then visit the patients and make the diagnoses.

### Outcomes

The main outcome was diagnosis recorded as either TIA, stroke or other diagnoses (i.e. non-TIA/stroke). TIA was defined as one or more brief episodes of neurological dysfunction resulting from focal cerebral ischaemia not associated with permanent cerebral infarction, lasting shorter than 24 h [22]. Stroke was defined as sudden onset of focal neurological deficit of vascular aetiology with CT/MRI evidence of acute focal ischaemia or haemorrhage [23]. Any other diagnosis which mainly comprised stroke/TIA mimics such as migraine and subdural haematoma etc. was classified under "other diagnoses". Four experienced stroke physicians assessed the patients and made the final diagnoses based on history, clinical findings, neuro-imaging and other relevant investigations (e.g. carotid Doppler).

### Statistical methods

Data were analysed using Stata version 10.1 (Stata- Corp, College Station, TX, USA). Student’s t-test was used to compare mean values of continuous variables and Pearson's χ^2^ test was used to compare proportions of outcomes in each of the orthostatic hypertension groups with the "no rise" group independently. We subsequently used a stepwise multivariate logistic regression approach to examine the association between blood pressure groups and diagnosis of cerebrovascular disease, transient ischemic attack, and stroke adjusting for confounding variables. Sensitivity analysis was used to assess for the effects of missing data.

## Results

### Participants and descriptive data

Among a total of 5311 patients in the cohort, we excluded 2110 participants due to missing data on BP measurements and age. Obesity and use of antihypertensive medications were significantly more common among patients in the included cohort compared to the excluded cohort (Complete comparison of included vs excluded cohort: Additional file 1: Table S1). Among the 3201 patients in the included cohort, 2344 were classified in the "no orthostatic hypertension", 167 in "systolic orthostatic hypertension", 776 in "diastolic orthostatic hypertension", and 86 in "combined orthostatic hypertension" groups. Moreover, 1123 patients were diagnosed with TIA, 658 with stroke and 1408 did not have any cerebrovascular diagnoses. Table 1 shows the variables in each of the study groups based on the dynamics of orthostatic blood pressure change.

**Table 1:** Characteristics for 3201 participants in TIA clinic stratified by no rise in blood pressure, rise in systolic blood pressure alone, diastolic blood pressure alone and rise of both systolic and diastolic blood pressure on standing

Variable	No orthostatic hypertension (n = 2344)	Systolic orthostatic hypertension (n = 167)	Diastolic orthostatic hypertension (n = 776)	Combined orthostatic hypertension (n = 86)
Age	73 (± 11) N = 2344	70 (± 13) N = 167	70 (± 12) N = 776	68 (± 13) N = 86
Male	1172/2344 (50%)	92/167 (55%)	401/776 (52%)	52/86 (60%)
Previous stroke	216/2344 (9%)	18/167 (11%)	60/776 (8%)	9/86 (10%)
Previous TIA	251/2344 (11%)	19/167 (11%)	70/776 (9%)	8/86 (9%)
Previous atrial fibrillation	275/2344 (12%)	18/167 (11%)	68/776 (9%)	7/86 (8%)
Previous hypertension	1309/2344 (56%)	88/167 (53%)	377/776 (49%)	43/86 (50%)
Previous ischemic heart disease	403/2344 (17%)	29/167 (17%)	113/776 (15%)	16/86 (19%)
Previous peripheral vascular disease	90/2344 (4%)	6/167 (4%)	24/776 (3%)	1/86 (1%)
Previous diabetes	341/2344 (15%)	27/167 (14%)	77/776 (10%)	12/86 (14%)
Obesity	587/2344 (25%)	51/167 (31%)	200/776 (26%)	27/86 (31%)
BMI	27 (± 5) N = 2262	27 (± 5) N = 163	27 (± 5) N = 761	28 (± 5) N = 83
Hyperlipidemia	806/2344 (34%)	61/167 (37%)	254/776 (33%)	30/86 (35%)
Smoker (current or ex-smoker)	1376/2344 (59%)	105/167 (63%)	473/776 (61%)	55/86 (64%)
Alcohol consumption	225/2344 (10%)	23/167 (14%)	96/776 (12%)	16/86 (19%)
Thiazide use	421/2344 (18%)	25/167 (15%)	122/776 (16%)	11/86 (13%)
ACEi or ARB use	839/2344 (36%)	58/167 (35%)	248/776 (32%)	29/86 (34%)
Beta blocker use	467/2344 (20%)	32/167 (19%)	137/776 (18%)	16/86 (19%)
Diuretic use	268/2344 (11%)	15/167 (9%)	67/776 (9%)	6/86 (7%)
Calcium channel blocker use	432/2344 (18%)	18/167 (11%)	115/776 (15%)	12/86 (14%)
Alpha blocker use	146/2344 (6%)	9/167 (5%)	26/776 (3%)	4/86 (5%)
Diastolic BP standing	78/2344 (± 13)	85 (± 13) N = 167	88 (± 12) N = 776	90 (± 13) N = 86
Systolic BP standing	146 (± 26) N = 2344	171 (± 26) N = 167	152 (± 25) N = 776	171 (± 26) N = 86
Diastolic BP lying	78 (± 14) N = 2344	73 (± 12) N = 167	71 (± 12) N = 776	72 (± 11) N = 86
Systolic BP lying	154 (± 26) N = 2344	142 (± 24) N = 167	147 (± 24) N = 776	142 (± 25) N = 86
Diagnosis				
TIA	855 (37%)	52 (31%)	234 (30%)	18 (21%)
Stroke	484 (21%)	29 (17%)	161 (21%)	16 (19%)
Other	994 (43%)	86 (52%)	380 (49%)	52 (60%)

Presented in the table are mean and standard deviation for continuous variables and number and percent for categorical variables

*BP* blood pressure, *TIA* transient ischemic attack

### Outcome data

Odds of cerebrovascular disease were lower among patients with combined systolic and diastolic orthostatic hypertension compared to patients without orthostatic hypertension. This correlation persisted when four different models were employed to adjust for confounding variables including age, sex, smoking, history of stroke, transient ischemic attack, known risk factors for cerebrovascular disease and use of certain antihypertensive medications (Table 2). However, patients with isolated systolic or diastolic orthostatic hypertension did not show a significant difference in the odds of cerebrovascular disease even when adjusted for potential confounding variables (Table 2).

**Table 2:** Stepwise multivariate logistic regression to examine the association between blood pressure groups and diagnosis of cerebrovascular disease, transient ischemic attack and stroke

Outcomes/level of adjustment	Systolic orthostatic hypertension compared to No orthostatic hypertension		Diastolic orthostatic hypertension compared to No orthostatic hypertension		Combined systolic and diastolic orthostatic hypertension compared to No orthostatic hypertension	
	n	OR (95% CI)	n	OR (95% CI)	n	OR (95% CI)
Odds of CVD						
Model A	3201	0.79 (0.57–1.08)	3201	0.87 (0.73–1.02)	3201	0.55 (0.35–0.87)
Model B	3103	0.79 (0.57–1.09)	3103	0.89 (0.75–1.06)	3103	0.56 (0.35–0.89)
Model C	3103	0.79 (0.57–1.09)	3103	0.89 (0.75–1.05)	3103	0.56 (0.36–0.90)
Model D	3103	0.79 (0.57–1.09)	3103	0.89 (0.75–1.05)	3103	0.56 (0.35–0.89)
Odds of stroke						
Model A	3201	0.83 (0.55–1.26)	3201	1.08 (0.88–1.32)	3201	0.93 (0.53–1.62)
Model B	3103	0.82 (0.54–1.25)	3103	1.11 (0.90–1.36)	3103	0.90 (0.51–1.59)
Model C	3103	0.83 (0.54–1.26)	3103	1.11 (0.91–1.37)	3103	0.90 (0.51–1.59)
Model D	3103	0.83 (0.43–1.26)	3103	1.11 (0.90–1.36)	3103	0.89 (0.50–1.58)
Odds of TIA						
Model A	3201	0.87 (0.62–1.22)	3201	0.81 (0.68–0.96)	3201	0.51 (0.30–0.87)
Model B	3103	0.87 (0.62–1.23)	3103	0.82 (0.68–0.98)	3103	0.54 (0.32–0.92)
Model C	3103	0.87 (0.62–1.23)	3103	0.81 (0.68–0.98)	3103	0.54 (0.32–0.93)
Model D	3103	0.87 (0.62–1.24)	3103	0.82 (0.68–0.98)	3103	0.54 (0.32–0.93)

Model A: adjusted for age and sex

Model B: adjusted for Model A and smoking, alcohol consumption, body mass index

Model C: adjusted for Model B and previous stroke, transient ischemic attack, atrial fibrillation, hypertension, ischemic heart disease, peripheral vascular disease, diabetes and hyperlipidemia

Model D: adjusted for Model C and use of thiazide, ACE inhibitor or ARB, beta-blocker, diuretic, calcium channel blocker and alpha blocker

The multivariate logistic regression analysis revealed a stronger association between combined systolic and diastolic orthostatic hypertension with lower rates of cerebrovascular disease among patients with obesity (OR 0.31 95% CI 0.12–0.80) and non-smokers (OR 0.34 95% CI 0.15–0.80) compared to normal-weight people (OR 0.74 95% CI 0.42–1.28) and smokers (OR 0.73 95% CI 0.41–1.29) respectively. Also, the odds of a cerebrovascular disease diagnosis decreased more sharply in normotensive patients (OR 0.42 95% CI 0.19–0.92) with a rise in both systolic and diastolic blood pressure upon standing compared to patients with underlying hypertension (OR 0.64 95% CI 0.36–1.15) with the same orthostatic BP response (Table 3).

**Table 3:** Multivariate logistic regression to examine the association between blood pressure groups and diagnosis of cerebrovascular disease, transient ischemic attack and stroke stratified by age, smoking status, hypertension, use of antihypertensive medications, obesity and peripheral vascular disease

Outcomes/level of adjustment	Rise in systolic BP compared to no rise		Rise in diastolic BP compared to no rise		Rise in both systolic and diastolic BP compared to no rise	
	n	OR (95% CI)	n	OR (95% CI)	n	OR (95% CI)
Odds of CVD						
Age ≤ 65 years	828	0.73 (0.40–1.31)	828	0.73 (0.53–1.00)	828	0.47 (0.21–1.03)
Age > 65 years	2275	0.85 (0.57–1.27)	2275	0.90 (0.73–1.11)	2275	0.69 (0.38–1.24)
Non-smoker	1255	0.87 (0.51–1.51)	1255	0.80 (0.61–1.05)	1255	0.34 (0.15–0.80)
Smoker	1848	0.77 (0.51–1.16)	1848	0.93 (0.74–1.15)	1848	0.73 (0.41–1.29)
No hypertension	1070	0.68 (0.39–1.18)	1070	0.88 (0.66–1.16)	1070	0.42 (0.19–0.92)
Hypertension	2033	0.84 (0.55–1.28)	2033	0.88 (0.70–1.09)	2033	0.64 (0.36–1.15)
No antihypertensive	1260	0.70 (0.43–1.16)	1260	0.85 (0.65–1.09)	1260	0.48 (0.24–0.98)
Antihypertensive	1843	0.85 (0.55–1.33)	1843	0.91 (0.72–1.14)	1843	0.63 (0.34–1.17)
No obesity	2303	0.87 (0.58–1.30)	2303	0.90 (0.74–1.10)	2303	0.74 (0.42–1.28)
Obesity	800	0.67 (0.37–1.22)	800	0.87 (0.62–1.22)	800	0.31 (0.12–0.80)
No PVD	2991	0.79 (0.57–1.11)	2991	0.89 (0.75–1.06)	2991	0.58 (0.36–0.92)
PVD	112	0.35 (0.04–2.81)	112	0.71 (0.25–2.05)	112	1 (no events)
Odds of stroke						
Age ≤ 65 years	828	1.42 (0.70–2.89)	828	0.71 (0.46–1.12)	828	1.30 (0.54–3.13)
Age > 65 years	2275	0.65 (0.38–1.10)	2275	1.23 (0.97–1.55)	2275	0.71 (0.33–1.53)
Non-smoker	1255	0.56 (0.25–1.25)	1255	1.03 (0.73–1.44)	1255	0.46 (0.14–1.55)
Smoker	1848	1.01 (0.61–1.66)	1848	1.15 (0.89–1.50)	1848	1.17 (0.60–2.27)
No hypertension	1070	0.68 (0.31–1.49)	1070	1.08 (0.74–1.56)	1070	0.92 (0.34–2.46)
Hypertension	2033	0.87 (0.52–1.44)	2033	1.11 (0.87–1.43)	2033	0.86 (0.42–1.74)
No antihypertensive	1260	0.85 (0.43–1.65)	1260	1.11 (0.80–1.55)	1260	1.10 (0.47–2.58)
Antihypertensive	1843	0.80 (0.46–1.37)	1843	1.10 (0.84–1.43)	1843	0.75 (0.34–1.63)
No obesity	2303	0.86 (0.52–1.42)	2303	1.10 (0.87–1.40)	2303	1.10 (0.57–2.13)
Obesity	800	0.83 (0.38–1.84)	800	1.12 (0.74–1.71)	800	0.60 (0.17–2.06)
No PVD	2991	0.82 (0.54–1.26)	2991	1.13 (0.92–1.40)	2991	0.91 (0.51–1.61)
PVD	112	0.91 (0.04–20.00)	112	0.32 (0.06–1.77)	112	1 (no events)
Odds of TIA						
Age ≤ 65 years	828	0.46 (0.21–1.01)	828	0.84 (0.59–1.19)	828	0.17 (0.04–0.73)
Age > 65 years	2275	1.12 (0.75–1.68)	2275	0.77 (0.62–0.95)	2275	0.86 (0.46–1.60)
Non-smoker	1255	1.22 (0.69–2.14)	1255	0.77 (0.57–1.03)	1255	0.45 (0.17–1.20)
Smoker	1848	0.74 (0.47–1.15)	1848	0.83 (0.66–1.04)	1848	0.60 (0.32–1.15)
No hypertension	1070	0.80 (0.45–1.43)	1070	0.83 (0.61–1.12)	1070	0.34 (0.13–0.91)
Hypertension	2033	0.92 (0.59–1.42)	2033	0.80 (0.64–1.00)	2033	0.67 (0.35–1.29)
No antihypertensive	1260	0.73 (0.43–1.26)	1260	0.78 (0.59–1.02)	1260	0.35 (0.14–0.86)
Antihypertensive	1843	1.00 (0.63–1.58)	1843	0.84 (0.66–1.06)	1843	0.73 (0.37–1.45)
No obesity	2303	0.95 (0.63–1.44)	2303	0.83 (0.68–1.03)	2303	0.65 (0.35–1.20)
Obesity	800	0.69 (0.35–1.36)	800	0.79 (0.55–1.15)	800	0.29 (0.08–0.99)
No PVD	2991	0.88 (0.62–1.26)	2991	0.81 (0.67–0.97)	2991	0.56 (0.32–0.95)
PVD	112	0.34 (0.04–2.84)	112	1.30 (0.43–3.89)	112	1 (no events)

Adjusted for age, sex, smoking, alcohol consumption, body mass index, previous stroke, transient ischemic attack, atrial fibrillation, hypertension, ischemic heart disease, peripheral vascular disease, diabetes, hyperlipidemia, use of thiazide, ACE inhibitor or ARB, beta-blocker, diuretic, calcium channel blocker and alpha blocker. Note that for analysis of a particular variable group, the adjustments were made for all variables except for the particular variable

*CVD* cerebrovascular disease (stroke or TIA), *PVD* peripheral vascular disease, *TIA* transient ischemic attack

Postural rise in blood pressure was not significantly correlated with the diagnosis of stroke in any of the adjustment models. Nevertheless, the incidence of TIA was lower among patients with diastolic (OR 0.81 95% CI 0.68–0.96) and combined (OR 0.51 95% CI 0.30–0.87) types of orthostatic hypertension compared to patients without postural rise in blood pressure. The latter correlation was the strongest among patients with combined orthostatic rise in systolic and diastolic blood pressure especially when adjusted for age and sex. Further analysis showed that the odds of TIA associated with orthostatic BP rise were lower among the subgroup of patients younger than 65 years compared to older patients (OR 0.17 vs. 0.86), those without a history of HTN compared with individuals with a past history of hypertension (OR 0.34 vs. 0.67) and patients who did not take medications for hypertension compared to those who took medications for hypertension (OR 0.35 vs. 0.73) (Table 3).

Sensitivity analysis excluding patients with orthostatic hypotension from the control group showed consistent results with the primary findings (Additional file 1: Table S2).

## Discussion

We found that patients with combined OHT or isolated diastolic OHT were significantly less commonly diagnosed with TIA compared to patients without OHT. However, there was no association between OHT and the diagnosis of stroke. Patients with OHT were also less commonly diagnosed with cerebrovascular disease compared to patients without OHT. While orthostatic hypotension (OH) is a known risk factor for stroke and cerebrovascular diseases, the possible role of orthostatic hypertension has not been studied as well [24–26].

The findings from the ARIC study showed a higher rate of lacunar stroke among patients with orthostatic rise or fall in blood pressure compared to patient without orthostatic changes in BP [20]. Other studies have also pointed to the same findings in line with more advanced deep white matter lesions among these patients [9, 17, 27].

Interestingly, we found that OHT decreased the rate of TIA diagnoses but not stroke. This finding may be related to our study population at a TIA clinic which does not include major stroke patients who would refer to the emergency department. Moreover, incidence of TIA subtypes seems to vary in different regions of the world. One study evaluating TIA patients in Japan showed that the most common etiologic subtype was lacunar TIA accounting for 31% of total TIA cases and that these patients were at a higher risk of early recurrent stroke during admission [28]. We know that orthostatic hypertension has been proposed as a potential risk factor for lacunar stroke [20, 27]. These population-based differences in TIA subtypes and risk factors may be the reasons why the findings from our study and other studies from England showed a lower rate of cerebrovascular disease and especially TIA among patients with OHT whereas the reverse has been shown in studies on the Japanese population [8, 10, 16, 20, 29].

Another interesting area of research is the association between orthostatic changes in blood pressure and circadian BP patterns. Kario et al. proposed that orthostatic hypertension is associated with extreme nocturnal dipping of blood pressure [30]. This pattern of diurnal blood pressure variation was found to be associated with silent cerebral infarcts, a higher incidence of cerebral ischemia during the night and an elevated risk of haemorrhagic stroke in the morning due to exaggerated morning BP surge [30–36]. Two other studies also found that extreme night-time dipping of blood pressure was associated with silent cerebral infarcts [35, 37]. The association between OH and reverse-dipping blood pressure at night has been widely studied and shown to be associated with increased risk of cardiovascular events [38]. The rate of cardiovascular events seems to increase with both extreme-dipping and reverse-dipping patterns of diurnal blood pressure variation [38]. Whether OHT and extreme night-time dipping -that seem to coexist in many patients-constitute a distinct pattern of BP variation associated with cerebrovascular clinical outcomes needs further clarification in prospective studies.

In our study, the association between OHT and lower rate of TIA diagnosis was significantly stronger in patients younger than 65 years. Although OHT has been found more commonly among patients with hypertension, the association between OHT with lower rate of TIA diagnosis was stronger among non-hypertensive patients or individuals who weren't using antihypertensive medications in the current study [5, 8, 9, 19, 35, 39–41]. In one study, where OHT was associated with an increase in all-cause mortality among elderly patients, CVD-related mortality was found more commonly among the subgroup of patients with OHT who did not have HTN, heart failure, CAD, or AF [7]. Another study that included older institutionalized patients indicated that OHT increased cardiovascular morbidity and mortality independent of sitting hypertension [4]. Most previous studies focusing on patients with orthostatic hypertension have only included the elderly [7, 8, 35]. Most of these studies point to increased rates of cerebrovascular pathology, mortality, impaired cognition, and frailty in older patients with OHT [4, 6, 7, 16, 42–44]. These findings in line with our results support the notion that while orthostatic hypertension may be a protective factor for cerebral ischemia and TIA in the short-term, it may be a precursor to hypertension that in turn increases the risk of cerebrovascular diagnoses in the long-term [7, 10, 45].

The findings from a post-mortem study of stroke patients show that the mechanism underlying the association between cerebrovascular disease and OHT may be severe carotid baroreceptor damage associated with severe carotid atheromatosis in ischemic stroke patients [13]. One study that specifically aimed to study the hemodynamics of postural changes in BP found that patients with OHT have a significantly more prominent increase in heart rate and systemic vascular resistance upon standing compared to other groups of hypertensive patients [37]. The study conducted by Petersen et al. shows that cardiac sympathetic input and cerebrovascular resistance were higher among young people with symptomatic transient OHT compared to symptomatic and asymptomatic controls [39].

A review of cases who had undergone carotid body resection shows that these patients have increased levels of BP variability associated with unopposed sympathetic activation [46]. Increased peripheral arteriolar constriction among patients with OHT has also been proposed as a pathogenic mechanism in OHT [47]. These pathogenic mechanisms point to an increased risk of cerebrovascular disease associated with OHT via increased vascular stress in the long-term. However, it is still unclear how the increase in standing BP especially in individuals with normal sitting blood pressure would affect the event of a stroke or TIA. Although we did not specifically aim to assess this issue in our study, our findings that point to a protective effect of OHT in TIA may provide some insight.

Our study should be interpreted considering the following limitations: We could not assess for the direction of the association due to the retrospective nature of data analysis and the observational design of our study. Although we adjusted for major known confounding variable in the analysis, observational studies are also limited by potential confounding from measured and unmeasured confounders. Moreover, a causal relationship cannot be inferred from the results. The control group included patients with orthostatic hypotension (OH) as well as patients with normal orthostatic BP dynamics; since OH may itself be associated with increased risk of cerebrovascular pathology, this may have introduced bias. Although, sensitivity analysis showed that excluding patients with postural hypotension did not affect the findings. Readers should also keep in mind that our study was carried out in a TIA clinic and the results cannot be generalized to the entire spectrum of patients with cerebrovascular disease; specifically, patients with severe and acute symptoms usually refer to emergency department and were not part of this study cohort. Thus, patients with major stroke who would refer to the emergency department were not assessed in our study and we cannot judge the effects of OHT on major stroke based on our findings.

We recommend further research on this topic with a prospective design including a cohort of individuals with and without hypertension at various ages to compare the short-term and long-term effects of OHT on the incidence of cerebrovascular disease in relation to age and sitting BP.

In conclusion, our study showed an association between combined and isolated diastolic orthostatic hypertension with a lower rate of TIA diagnosis and cerebrovascular disease but not stroke. These associations were stronger among patients younger than 65 years and without a history of diagnosed hypertension. It is possible that orthostatic hypertension is a protective factor for TIA among younger normotensive patients.

## Supplementary Information

**Additional file 1.** Comparison between the paticipants’ characteristics between the included and the excluded cohort.

## Data Availability

The supporting data of these findings are available upon reasonable request to the corresponding author.
