# Supplementary material for: Prevalence of orthostatic hypertension and its association with cerebrovascular diagnoses in patients with suspected TIA and minor stroke
Source: BMC Cardiovasc Disord. 2022 Apr 9;22:161. doi: 10.1186/s12872-022-02600-1 (PMC8994299; doi:10.1186/s12872-022-02600-1)
Supplement: Supplementary file 1 — Additional file 1. Comparison between the paticipants’ characteristics between the included and the excluded cohort. [file 12872_2022_2600_MOESM1_ESM.docx]

**Supplementary Table 1:** Table comparing the participant characteristics of the included and excluded cohort

| Variable | Included cohort (n=3201) | Excluded cohort (n=2110) | p-value* |
| --- | --- | --- | --- |
| Age | 72 (±11) N=3201 | 73 (±9) N=2100 | <0.001 |
| Male | 1613/3201 (50%) | 1080/2110 (51%) | 0.57 |
| Previous stroke | 285/3201 (9%) | 183/2110 (9%) | 0.77 |
| Previous TIA | 332/3201 (10%) | 284/2110 (13%) | 0.001 |
| Previous atrial fibrillation | 354/3201 (11%) | 200/2110 (9%) | 0.07 |
| Previous hypertension | 1731/3201 (54%) | 1173/2110 (56%) | 0.28 |
| Previous ischemic heart disease | 529/3201 (17%) | 365/2110 (17%) | 0.46 |
| Previous peripheral vascular disease | 119/3201 (4%) | 136/2110 (6%) | <0.001 |
| Previous diabetes | 430/3201 (13%) | 249/2110 (12%) | 0.08 |
| Obesity | 811/3201 (25%) | 34/2110 (2%) | <0.001 |
| BMI | 27 (±5) N=3103 | 28 (±5) N=81 | 0.30 |
| Hyperlipidemia | 1091/3201 (34%) | 589/2110 (28%) | <0.001 |
| Smoker (current or ex-smoker) | 1899/3201 (59%) | 1268/2110 (60%) | 0.58 |
| Alcohol consumption | 328/3201 (10%) | 220/2110 (10%) | 0.83 |
| Thiazide use | 557/3201 (17%) | 28/2110 (1%) | <0.001 |
| ACEi or ARB use | 1116/3201 (35%) | 50/2110 (2%) | <0.001 |
| Beta blocker use | 620/3201 (19%) | 21/2110 (1%) | <0.001 |
| Diuretic use | 344/3201 (11%) | 20/2110 (1%) | <0.001 |
| Calcium channel blocker use | 553/3201 (17%) | 29/2110 (1%) | <0.001 |
| Alpha blocker use | 177/3201 (6%) | 8/2110 (0.4%) | <0.001 |
| Diastolic BP standing | 80 (±13) N=3201 | 80 (±25) N=49 | 0.94 |
| Systolic BP standing | 148 (±26) N=3201 | 140 (±29) N=51 | 0.03 |
| Diastolic BP lying | 77 (±14) N=3201 | 76 (±12) N=51 | 0.69 |
| Systolic BP lying | 152 (±26) N=3201 | 144 (±32) N=58 | 0.02 |
| Diagnosis  TIA  Stroke  Other | 1123 (35%)  658 (21%)  1408 (44%) | 976 (47%)  531 (25%)  591 (28%) | <0.001 |

Exclude cohort consists of participants with missing data.

BP=blood pressure, TIA=transient ischemic attack. Presented in the table are mean and standard deviation for continuous variables and number and percent for categorical variables.

*T-test was used to compare continuous variables between missing and non-missing cohort. Chi^2^ test to compare categorical variables between missing and non-missing cohort

**Supplementary Table 2:** Sensitivity analysis of stepwise multivariate logistic regression to examine the association between blood pressure groups and diagnosis of cerebrovascular disease, transient ischemic attack and stroke with participants with postural hypotension excluded

| Outcomes/level of adjustment | Rise in systolic BP compared to no rise | | Rise in diastolic BP compared to no rise | | Rise in both systolic and diastolic BP compared to no rise | |
| --- | --- | --- | --- | --- | --- | --- |
|  | n | OR (95% CI) | n | OR (95% CI) | n | OR (95% CI) |
| Odds of CVD  Model A | 2594 | 0.77 (0.55-1.07) | 2594 | 0.89 (0.74-1.06) | 2594 | 0.55 (0.35-0.87) |
| Odds of stroke  Model A | 2594 | 0.81 (0.53-1.24) | 2594 | 1.14 (0.92-1.42) | 2594 | 0.95 (0.55-1.66) |
| Odds of TIA  Model A | 2594 | 0.86 (0.61-1.22) | 2594 | 0.80 (0.66-0.96) | 2594 | 0.50 (0.29-0.85) |

Model A: adjusted for age and sex
